# Long-term cancer risk after kidney transplantation: a German primary care cohort study

**DOI:** 10.1038/s41416-025-03153-8

**Published:** 2025-08-08

**Authors:** Karel Kostev, Bernhard MW Schmidt, Zhejia Tian

**Affiliations:** 1 University Hospital, The Philipps University of Marburg, Marburg, Germany; 2 IQVIA, Epidemiology, Frankfurt am Main, Germany; 3 Department of Nephrology and Hypertension, Hannover Medical School, Hannover, Germany

**Keywords:** Outcomes research, Risk factors, Cancer epidemiology

We read with great interest the nationwide Scottish registry linkage study by Nimmo et al. (*N* = 4033 kidney transplant recipients [KTRs]; 1997–2021), which reported a nearly fourfold increased overall cancer risk compared to the general population (standardised incidence ratio [SIR] 3.9; 95% CI 3.7–4.2), with particularly high elevations in non-melanoma skin cancer, lymphoma, and kidney cancer [1]. Importantly, younger recipients (<40 years) experienced an even more pronounced relative risk—approximately sevenfold—underscoring age-related vulnerability.

We wish to report complementary findings from Germany—a multicentre cohort of 2302 first-time adult KTRs transplanted between 2005 and 2023. Our study utilised electronic medical records from the IQVIA™ Disease Analyzer database, which includes anonymised demographic variables, diagnoses, and prescriptions of outpatients recorded in private practices [2]. We included patients aged ≥18 years with a first recorded renal transplantation (ICD-10: Z94.0) in 910 general practices between January 2005 and December 2023, excluding those with prior or concurrent cancer diagnoses. Ten-year cancer incidence (only the first malignancy) was analysed using Kaplan–Meier curves, and univariable Cox regression was applied to assess the association between renal transplantation and subsequent cancer. Patients were matched 1:5 to non-transplanted individuals using nearest-neighbour propensity score matching based on sex, age, index year, and comorbidities (obesity, diabetes, hypertension, dyslipidaemia, nicotine and alcohol addiction, and chronic obstructive pulmonary disease).

A total of 13,812 individuals were included (2302 with renal transplant and 11,510 without). The mean age was 54.0 years (SD: 14.9), and 42.3% were female. The cumulative 10-year cancer incidence was 23.3% among transplant recipients and 11.1% in the matched control group (*p* < 0.001). Renal transplantation was associated with a significantly increased overall cancer risk (Hazard Ratio [HR]: 2.13; 95% CI: 1.82–2.49), with stronger associations in men (HR: 2.29; 95% CI: 1.88–2.78) than women (HR: 1.89; 95% CI: 1.47–2.45), and in younger patients (≤50 years: HR: 2.99; 95% CI: 2.07–4.32) compared to older patients (>60 years: HR: 2.09; 95% CI: 1.69–2.59) (Table 1).

**Table 1:** Association between renal transplantation and 10-year-incidence of cancer diagnoses in primary care patients.

Cancer type	HR (95% CI)	*p* value
Cancer total	2.19 (1.82–2.49)	<0.001
Renal cancer	6.63 (3.22–13.66)	<0.001
Non-melanoma skin cancer	6.23 (4.40–8.83)	<0.001
Lymphomas	3.86 (2.48–6.01)	<0.001
Melanoma	1.58 (0.62–4.04)	0.339
Colorectal cancer	1.46 (0.77–2.76)	0.242
Prostate cancer	1.35 (0.77–2.38)	0.299
Lung cancer	1.22 (0.66–2.26)	0.531
Leukaemia	0.88 (0.19–4.09)	0.874
Bladder cancer	0.75 (0.17–3.36)	0.702

In line with Nimmo et al. [1], renal cancer (HR: 6.63; 95% CI: 3.22–13.66), non-melanoma skin cancer (HR: 6.23; 95% CI: 4.40–8.83), and lymphomas (HR: 3.86; 95% CI: 2.48–6.01) (Table 1) were the malignancies most strongly associated with transplantation. No significant associations were observed for common cancers such as lung, colorectal, and prostate cancer. Our findings corroborate the Scottish data, highlighting increased post-transplant cancer incidence—particularly in male and younger recipients—and a consistent pattern of elevated site-specific risks.

Methodologically, our German cohort differs from the Scottish study in two key respects: (1) comparison to a matched non-transplanted cohort rather than the general population, and (2) use of hazard ratios instead of standardised incidence ratios. Despite these differences, the directional consistency across studies strengthens the evidence base. Limitations of our analysis include lack of data on smoking, ethnicity, sun exposure, and socioeconomic factors. Additionally, data were limited to outpatient primary care records and did not include specialist or hospital-based diagnoses.

Our findings affirm a substantially increased long-term cancer risk among kidney transplant recipients, especially for renal, non-melanoma skin cancers, and lymphomas. The elevated risk in younger and male patients suggests the need for tailored, risk-stratified post-transplant surveillance strategies. Clinicians should maintain heightened vigilance for malignancy in this population and consider proactive dermatological screening and oncologic evaluation as part of long-term transplant care. Future research should incorporate more comprehensive datasets to explore modifiable risk factors and evaluate the efficacy of targeted cancer prevention interventions in this high-risk group.

## Data Availability

The datasets generated during and/or analysed during the current study are not publicly available due to privacy restrictions but are available from the corresponding author on reasonable request.
